# Predictors of changes in incisor inclination during orthodontic levelling and alignment with fixed appliances: a retrospective cross-sectional study

**DOI:** 10.1186/s13005-025-00519-4

**Published:** 2025-05-26

**Authors:** Camilla Sahm, Christian Kirschneck, Peter Proff, Eva Paddenberg-Schubert

**Affiliations:** 1 Department of Orthodontics, University Hospital Regensburg, 93053 Regensburg, Germany; 2 Department of Orthodontics, University of Bonn, 53111 Bonn, Germany

**Keywords:** Lateral cephalogram, Tooth axis, Proclination, Torque, Personalized orthodontics, Treatment planning

## Abstract

**Introduction:**

Labial tipping of the incisors, observed during levelling and alignment in orthodontic treatment with multibracket-appliances, can be an undesired side effect due to its association with relapse and attachment loss in excessive cases. Therefore, its pre-treatment prediction is useful to individualise treatment plans correspondingly. This retrospective cross-sectional study aimed to establish regression equations predicting incisors’ inclination changes during levelling and alignment with fixed appliances in orthodontic patients using lateral cephalograms. Potential predictors analysed included clinical and cephalometric parameters.

**Methods:**

Patients of any age and malocclusion were screened for inclusion, and the upper and lower arches were evaluated separately. Lateral cephalograms taken at T0 and T1, initial plaster models and patient records were analysed. Multiple linear regression models were performed to establish regression equations, identifying predictors of incisors’ inclination changes.

**Results:**

The final study population was comprised of 216 female (*n* = 123, 56.9%) and male (*n* = 93, 43.1%) orthodontic patients (147 upper, 151 lower arches) aged between 9.3 and 30.0 years with a mean age of 13.1 years ± 2.1. Interrater- and intrarater-reliability showed perfect measurement concordance for all cephalometric parameters and moderate to perfect concordance for categorical variables. Sagittal changes of the upper incisors’ inclination (Δ1-NL) were dependent on initial anterior crowding, initial incisor inclination and intermaxillary elastics (corrected R^2^ = 0.375, *n* = 147). In the lower jaw, incisors’ proclination (Δ1-ML) was predicted by anterior crowding, incisor inclination, growth pattern, skeletal class and bracket type (corrected R^2^ = 0.468, *n* = 151). Changes in the interincisal angle (Δ1–1) were significantly predicted by mandibular anterior crowding and initial inclination of the upper and lower incisors (corrected R^2^ = 0.440, *n* = 82).

**Conclusions:**

Regression equations with specific clinical and cephalometric parameters are suitable to predict the degree of incisors’ inclination changes during alignment with fixed appliances. The amount of anterior crowding and the initial incisors’ inclination of the respective jaw were relevant in all three formulas. Using the predictors may reduce undesired excessive inclination changes and help in individualised treatment planning. However, as more than 50% of the variance are explained by other factors, they act as an adjunctive method to the remaining procedures of treatment planning.

**Supplementary Information:**

The online version contains supplementary material available at 10.1186/s13005-025-00519-4.

## Introduction

During orthodontic treatment with fixed appliances levelling and alignment is often accompanied by changes in the incisors’ inclination, especially by proclination [1]. Their excessive proclination is associated with relapse and attachment loss [2-6], potentially increasing sensitivity and risk of cavities and reducing aesthetics [2]. Furthermore, incisors’ inclination also affects facial attractiveness [7], with variations between different facial patterns [8, 9]. Therefore, variables predicting the changes in the incisors’ inclination appear promising to avoid functional and aesthetic undesired positions and inclinations of the front teeth. Knowing the expected proclination in advance allows the orthodontic practitioner for individualising the treatment plan accordingly, e.g. by considering tooth extractions to avoid excessive proclination in crowding cases.

Several factors could affect incisors’ inclination. Resolving crowding without tooth extraction, distalising or interproximal stripping can lead to an increase in arch length and incisors’ proclination [1, 10]. The association between proclination and the amount of crowding is well described [11-15]. For example, Fleming et al. identified a significant association between the degree of proclination during alignment and the initial space discrepancy [16]. Another potential predictor is Angle class, which describes the sagittal relation of reference teeth. For example, an evaluation of pre- to post-treatment changes in mesio-distal angulation of canines and first molars mostly did not differ between the various skeletal and Angle classes [17]. Furthermore, pre-treatment diagnostics often include an analysis of lateral cephalograms, which also evaluates skeletal class, i.e. the antero-posterior relation between the upper and lower jaw, growth pattern, the shape of the mandibular symphysis according to Björk and lip thickness. Comparing pre- to post-treatment cephalometrics, Kau and Bakos presented significant differences in the inclination of the lower incisors between skeletal class I, II and III [18]. Another routinely performed potential predictor is the three-dimensional analysis of dental casts, including the assessment of overbite. For example, deep bite correction can be associated with incisors’ proclination [19]. In addition to the above-mentioned variables, orofacial dysfunctions and habits are routinely assessed before orthodontic treatment. They can influence the inclination of the incisors before [20] and after treatment [21]. Also, different treatment strategies of missing teeth, i.e., space closure or opening, could affect the inclination [22] and mesiodistal angulation [23] resulting from orthodontic treatment.

Orthodontic treatment with fixed appliances can be performed with conventional TWIN-brackets or self-ligating brackets (SLB). A potential, although highly discussed advantage of SLB, is the reduction in friction between the arch wire and bracket slot [24, 25]. Some studies reported incisors’ proclination during alignment with both conventional brackets and SLB in the lower [26] and upper jaw [16]. In contrast, Celikoglu et al. did not observe differences in incisors’ proclination between the two bracket types when resolving crowding [1].

So far, the evidence available focusses on the correlation between incisors’ inclination changes and single other parameters, such as crowding or skeletal class. However, to the best of our knowledge, there is no investigation, which considered multiple clinical and cephalometric parameters as predictors of incisors’ proclination during alignment with fixed appliances. Furthermore, many of the existing studies evaluated pre- to post-treatment changes in only one arch. Hence, the aim of this retrospective cross-sectional study is to identify variables, which predict changes in incisors’ inclination during levelling and alignment with fixed appliances in orthodontic patients by comparing incisors’ inclination before (T0) and after alignment (T1) using lateral cephalograms. Potential predictors analysed included the bracket type (conventional vs. SLB), various cephalometric parameters (growth pattern, form of the symphysis, skeletal class, lip thickness, initial incisor inclination), forms of malocclusion (open vs. deep bite, initial anterior crowding, Angle class), functional analyses (orofacial dysfunction, habits) and specific auxiliary treatment devices (headgear, intermaxillary elastics). Therefore, this cross-sectional study tested the null hypothesis that neither cephalometric nor clinical variables predict the changes in inclination of the upper (1-NL) and lower incisors (1-ML) and of the interincisal angle (1-1) during alignment with multibracket-appliances in orthodontic patients.

## Methods

This study was conducted in accordance with the declaration of Helsinki and the approval of the ethical committee of the University of Regensburg, Germany (approval number 22-2822-104), and reported sticking to the STROBE guidelines.

**Table 1:** Inclusion and exclusion criteria applied in this study. T0 = before orthodontic treatment, T1 = after levelling and alignment

inclusion criteria	exclusion criteria
all age groups	cleft lip and palate or other craniofacial anomalies
male or female	syndromes
all ethnic groups	existing or previous severe pathologies (e.g. tumours, craniofacial fractures) in the cranial region
all types of malocclusion and dysgnathia except for cleft lip and palate and syndromes	no availability of a lateral cephalogram at T1 and T0 (at T0: with scale)
availability of lateral cephalograms at T0 and T1, with scale at T0	tooth extraction between T0 and T1 in the jaw considered
availability of patient records	specific treatment appliances or procedures (functional appliances, fixed class II mechanics, lingual arches, segmented arch mechanics, intrusion mechanics, pendulum or nance appliances, rectangular arch wires, hyrax or quadhelix appliances, open coils in the incisor region, Sweep or Anti-Spee arch wires, interproximal enamel reduction) in the jaw considered between T0 and T1

The retrospective epidemiological cross-sectional study was based on patients, who started orthodontic treatment and completed levelling and alignment between 2012 and 2022 at an orthodontic specialist practice in Bavaria, Germany.

The inclusion and exclusion criteria applied in this study are presented in Table 1. T0 was the examination before the start of orthodontic treatment, and T1 was defined as the end of levelling and alignment, equal to the treatment step before inserting a rectangular arch wire. The maximum time between T0 and T1 was two years. Therefore, this time interval was chosen to include patients, whose active treatment started delayed due to organis ational reasons. During the selection process, the upper and lower arch were analysed separately. The sample size was determined using the maximum number of appropriate patients within the period of recruitment of levelling and alignment.

Following anonymisation of the study participants, a separation between those, whose upper arch, and those, whose lower arch was included, was conducted. Further subgroups were built in order to distinguish between conventional brackets (TWIN) and SLB. The SLB used were either Speed (Hespeler Orthodontics Limited, Cambridge, Ontario, Canada) or Experience™ metal selfligature brackets (GC Orthodontics Europe, Breckerfeld, Germany). Conventional brackets were obtained from various manufacturers. All brackets had a slot size of 0.022 × 0.028 inch and the greatest wire dimension inserted was 0.018 inch, mostly made of NiTi.

Then, patient records between T0 and T1, including medical history, pre-treatment plaster models and clinical records as well as lateral cephalograms from T0 and T1, were analysed. All records were performed for medical reasons and not for the purpose of the study. Within the recruitment period, the x-ray device was changed from analogue to digital. Analogue X-ray images were digitised using the scanner Epson Expression 1600 Pro (Epson, Suwa, Nagano, Japan) and analysed with the software OnyxCeph3TM 3.2.230 Build 624 Polywell (Image Instruments, Chemnitz, Germany), as were the digital images. Analogue radiographs were conducted with the device OP 10E (Siemens Orthophos, Munich, Germany) and digital images were obtained using Planmeca ProMax 3D Mid/ FRS (Planmeca, Helsinki, Finland). In analogue images a voltage of 69 kV, a current of 15 mA and an exposure time of 0.64 s was applied, whereas in digital images the respective values were 70 kV, 16 mA and 10.5 s.

Lateral cephalograms, initial plaster models and the other records were analysed by one rater (CS). Prior to the main investigation, 50 randomly chosen patients were used to evaluate interrater- and intrarater-reliability for cephalometric analysis, classification of Angle class and determination of anterior crowding. The time interval of the repeated measurements (CS) for intrarater-reliability was two weeks. Interrater-reliability was assessed by two independent raters (CS, orthodontic specialist).

**Fig. 1:**
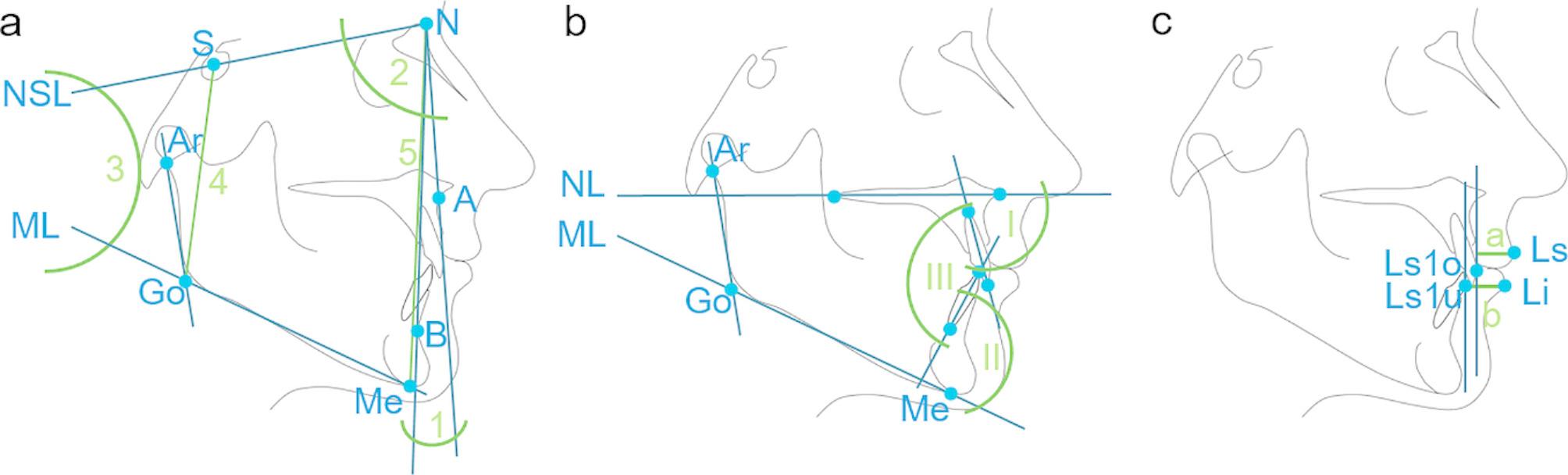
Relevant cephalometric **(a)** skeletal, **(b)** dental and **(c)** soft-tissue parameters. 1 = ANB-angle, 2 = SNA-angle, 3 = ML-NSL-angle, 4 = posterior facial height, 5 = anterior facial height, I = 1-NL, II = 1-ML, III = 1–1, a = upper lip thickness, b = lower lip thickness. Green lines/ angles indicate the site of the measurement, blue lines/ points represent parameters and references used to measure the parameters

The evaluated predictors were bracket type, initial anterior crowding in the upper and lower jaw, form of the symphysis, skeletal class, Angle class, growth pattern, form of malocclusion (overbite), lip thickness, orofacial dysfunction/ habits and specific auxiliary treatment devices (headgear, intermaxillary elastics). All relevant cephalometric parameters are explained in Fig. 1; Table 2.

**Table 2:** Definition and explanation of the relevant cephalometric parameters

cephalometric variable	definition	explanation
SNA [°]	angle between the line NA and the anterior cranial base (SN = NSL)	degree of maxillary prognathism
ML-NSL [°]	angle between the mandibular plane (ML) and NSL	inclination of the mandible
ANB [°]	angle between the lines NA an NB	skeletal class
individualised ANB [27] [°]	= -35.16 + 0.4×SNA + 0.2×ML-NSL	individualised norm value for skeletal class
Jarabak ratio [%]	ratio between posterior and anterior facial height (= (SGo/NMe) x 100)	growth pattern
1-NL [°]	angle between the axis of the most prominent upper incisor and the nasal line (NL)	inclination of the upper incisors
1-ML [°]	angle between the axis of the most prominent lower incisor and ML	inclination of the lower incisors
1–1 [°]	angle between the axes of most prominent upper and lower incisors	interincisal angle
upper lip thickness [mm]	linear distance of a perpendicular line from the most anterior point of the upper lip (Ls) to a vertical line parallel to the image edge passing through the labial contour of the most prominent upper incisor (Ls1o)	thickness of the upper lip
lower lip thickness [mm]	linear distance of a perpendicular line from the most anterior point of the lower lip (Li) to a vertical line parallel to the image edge passing through the labial contour of the most prominent lower incisor (Ls1u)	thickness of the lower lip

Regression equations were established for the three outcome variables changes in the inclination of the upper (∆1-NL) and lower incisors (∆1-ML) and the inclination of the incisors to each other (∆1–1).

Plaster models from T0 were used to evaluate initial anterior crowding, Angle class and form of malocclusion (open or deep bite). By means of clinical records, information concerning swallowing patterns, speech disorder, mouth breathing and habits was gathered. Furthermore, clinical records were analysed to detect the usage of specific auxiliary treatment devices (headgear, intermaxillary elastics).

Statistical analyses were conducted using the software IBM^®^ SPSS^®^ Statistics 20 (IBM, Armonk, NY, USA). In general, the method used to identify significant predictors of or correlations among specific variables (here changes in 1-NL, 1-ML, 1–1) is well reported in the literature [28-30]. First, interrater- and intrarater-reliability testing were performed, using Lin’s Concordance Correlation Coefficient (CCC) for all metric measurements and Cohen`s kappa (к) for all categorical variables [31, 32]. Then, applying multiple linear regression analyses, regression equations were established to identify predictors of the inclination changes of lower (Δ1-ML) and upper incisors (Δ1-NL) and of the interincisal angle (Δ1–1). Only parameters presenting sufficient interrater- and intrarater-reliability (CCC ≥ 0.8) and no collinearity with other predictor variables (Variance Inflation Factor (VIF) < 10, tolerance > 0.1) were considered in these regression analyses. Normal distribution of the data was assumed due to the central limit theorem. With ANOVA, the correlation coefficient R of the regression model was tested towards a significant difference to zero. Considering the regression coefficients, their statistical significance was tested (one sample test against zero) and the corresponding standard error and 95% confidence iterval (CI) were determined. The standard error and the 95% confidence interval were calculated for all regression coefficients. The significance level (α-error) was set at *p* ≤ 0.05.

## Results

**Fig. 2:**
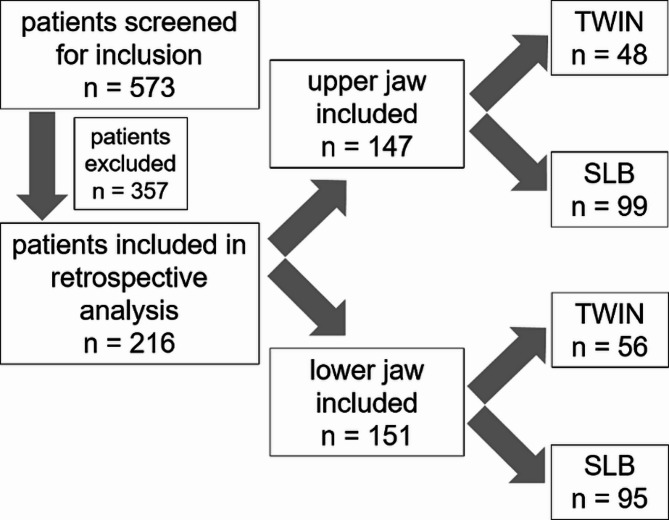
Flow-chart of the study population. TWIN = conventional bracket, SLB = self-ligating bracket

After checking inclusion and exclusion criteria, 216 patients were finally included in the study (Fig. 2). They were aged between 9.3 and 30.0 years with a mean age of 13.1 years ± 2.1. The study population was comprised of 123 female (56.9%) and 93 male (43.1%) subjects.

Upper and lower jaws were evaluated separately. In 69 (31.9%) subjects only the lower jaw and in 65 (30.1%) patients only the upper jaw was included. In the remaining 82 subjects (38.0%), both jaws were included. Among the upper arches, 99 patients (45.8%) were treated with SLB and 48 patients (22.2%) with conventional TWIN brackets. Similarly, in the lower jaw, 95 patients (44.0%) presented SLB and 56 subjects (25.9%) conventional TWIN brackets.

The average interval between the first and second lateral cephalogram was 12.5 months with a minimum of 5 months and a maximum of 24 months. Descriptive statistics of the baseline characteristics of the study population are presented in an additional table file (see Additional file 1).

Overall, between T0 and T1, i.e. during levelling and alignment, upper incisors proclined 3.59 ° on average. For lower incisors, a mean proclination of 2.07 ° was observed.

According to the perfect concordance (CCC ≥ 0.9) and very good correlation (κ 0.81–1.0), growth pattern (Jarabak ratio), 1–1, 1-NL, 1-NSL, 1-ML, upper and lower lip thickness, initial anterior space discrepancy in the upper and lower jaw and Angle class proofed reliable assessment. The form of the symphysis (Björk) showed a very good correlation for the interrater-reliability and a good correlation for the intrarater-reliability (κ 0.61–0.8).

Regression analyses were conducted with the dependent variables ∆1-NL, ∆1-ML and ∆1–1.

Based on the results of 147 patients, the change in the upper incisors’ inclination during alignment (∆1-NL) can be significantly predicted by the variables anterior crowding in the upper jaw, 1-NL and class II elastics, achieving a corrected R² of 0.375: *F* (3,143) = 30.197; *p* < 0.001.

∆1-NL [°] = -27.692 + 0.64 x anterior crowding _upper jaw_ + 0.455 × 1-NL − 1.364 x intermaxillary elastics (2 for class II elastics) (corrected R^2^ = 0.375).

Analysing 151 patients, the regression equation revealed that bracket type, anterior crowding in the lower jaw, ANB, individualised ANB, Jarabak ratio and 1-ML significantly predict the proclination of lower incisors (∆1-ML) during alignment: *F* (6,144) = 22.996; *p* < 0.001.

∆1-ML [°] = -55.308–1.856 x bracket-type _lower jaw_ [1 for SLB. 2 for TWIN] + 1.581 x anterior crowding _lower jaw_ [mm] + 0.825 x ANB [°] − 1.334 x individualised ANB [°] + 0.334 x Jarabak ratio [%] + 0.458 × 1-ML [°] (corrected R^2^ = 0.468).

Using the records of 82 patients, a regression formula was established for the change in the interincisal (∆interincisal angle). The variables anterior crowding in the lower jaw, 1-NL and 1-ML significantly predict the change in the interincisal angle: *F* (3,78) = 22.184; *p* < 0.001.

∆interincisal angle [°] = -54.230 + 4.284 x anterior crowding _lower jaw_ [mm] + 0.441 × 1-NL [°] + 0.307 × 1-ML [°] (corrected R^2^ = 0.440).

The summarised results of the multiple linear regression analyses for the three dependent variables are presented in Table 3.

**Table 3:** Summary of the significant predictors for each of the three dependent variables according to multiple regression analysis

outcome variable	variable	regression coefficient	standard error	95% CI	*p*-value
∆1-NL	anterior crowding upper jaw	0.640	0.282	0.083;1.196	0.025
	1-NL	0.455	0.055	0.347;0.564	< 0.001
	intermaxillary elastics (class II elastics)	-1.364	0.661	0.170;0.371	0.041
	regression coefficient	-27.692	3.772	-35.147;-20.236	< 0.001
∆1-ML	bracket type lower jaw	-1.856	0.664	-3.169;-0.542	0.006
	anterior crowding lower jaw	1.581	0.351	0.887;2.275	< 0.001
	ANB	0.825	0.219	0.393;1.257	< 0.001
	Individualised ANB	-1.334	0.307	-1.940;-0.728	< 0.001
	Jarabak ratio	0.334	0.082	0.172;0.496	< 0.001
	1-ML	0.458	0.069	0.321;0.595	< 0.001
	regression coefficient	-55.308	9.834	-74.746;-35.870	< 0.001
∆1–1	anterior crowding lower jaw	4.284	0.863	2.566;6.003	< 0.001
	1-NL	0.441	0.104	0.235;0.647	< 0.001
	1-ML	0.307	0.129	0.050;0.564	0.02
	regression coefficient	-54.230	12.751	-79.616;-28.845	< 0.001

## Discussion

The aim of this retrospective cross-sectional study was the identification of predictors of incisors’ inclination changes during levelling and alignment, using data from the regular initial diagnostics. Being able to predict the extent of proclination during alignment could lead to an adjustment of the individual treatment plan. Various cephalometric and clinical variables, taken routinely during pre-treatment diagnostics, were evaluated by multiple regression analyses to identify significant predictors and to establish regression equations for the expected changes in incisors’ inclination during alignment (Δ1-NL, Δ1-ML, Δ1–1). According to our results, inclination changes of the upper and lower incisors as well as of the interincisal angle can be predicted by various cephalometric and clinical parameters, so that the null hypothesis was rejected.

Concerning the expected proclination of the upper (Δ1-NL) and lower (Δ1-ML) incisors and the change in the interincisal angle (Δ1–1) during alignment, the regression equations established explained 37.5%, 46.8% and 44.0% of the total variance, respectively. Hence, more than 50% of the total variance are caused by other factors not considered in these formulas. Although these equations are helpful during treatment planning, they may only be used in addition to a complete diagnostic analysis.

Among the significant predictors, the baseline parameters anterior crowding and incisor inclination were positively correlated with the amount of proclination during levelling and alignment. Hence, the bigger anterior crowding and the steeper initial incisor inclination, the more proclination is to be expected. In line with this observation, the amount of space gain by incisors’ proclination was shown to depend on the initial incisors’ inclination, as the space gain becomes smaller with increased pre-treatment proclination [33]. Similarly to the positive correlation between incisor proclination and anterior crowding found in this study, Fleming et al. presented crowding as the main predictor of upper incisors’ proclination [16]. Furthermore, several studies reported an increase in mandibular incisors’ labial tipping during resolution of crowding [1, 14, 15, 34]. Of note, the effect of anterior crowding on the change in incisors’ inclination was smallest in the upper jaw, followed by the lower jaw, and biggest for the interincisal angle.

Concerning the predicting variable pre-treatment incisors’ inclination, all of the three regression equations included this parameter with a positive correlation. Few studies resemble our research. For example, Fleming et al. presented the initial inclination of the upper incisors to be associated with the post-alignment values [16]. Furthermore, Zimmer et al. described the post-treatment incisors’ inclination to be dependent on the pre-treatment inclination in most cases [35].

Class II elastics significantly predicted Δ1-NL with a negative correlation. Hence, the use of such elastics was accompanied by reduced proclination of the upper incisors. Similarly, Nelson et al. presented that class II elastics led to retroclination of maxillary incisors [36]. Moreover, class II elastics were reported to result in proclination of the lower incisors after treatment with fixed appliances in class II division 1 cases [37]. However, in our regression equation for the lower incisors, elastics did not serve as significant predictors. Differences in the observation period (levelling and alignment vs. complete treatment) and study population (all vs. class II division 1 cases) could explain this difference.

The growth pattern (Jarabak ratio) presented a positive association with the post-alignment inclination of the lower incisors in our equation. Hence, a more horizontal growth pattern leads to more proclination of the lower incisors. Similarly, Rozzi et al. observed a greater proclination of the incisors in low angle cases, which the authors explained by the levelling the curve of Spee without extrusion of the premolars due to strong vertical masticatory forces in patients with horizontal growth pattern [38]. Furthermore, Assi et al. found a positive correlation between the upper incisors’ inclination and horizontal patterns, especially after orthodontic treatment [39].

Regarding the lower incisors’ proclination, our results present a positive correlation with skeletal class (ANB angle), but a negative one with individualised ANB [27]. The latter in turn is positively associated with maxillary prognathism (SNA angle) and the mandible’s inclination (ML-NSL). According to the positive correlation with ANB, more proclination can be expected in skeletal class II, whereas in skeletal class III less proclination is predicted. Looking at the negative correlation of the individualised ANB, maxillary prognathism (SNA angle ↑) and clockwise rotation of the mandible (ML-NSL ↑) result in reduced proclination of the lower incisors during alignment. To the best of our knowledge, there are no studies available, which specifically examine these aspects. However, the positive correlation between ANB and lower incisors’ proclination has been described in the context of floating norms for an individual’s ideal incisors’ inclination, which describe more proclination of the lower and retroinclination of the upper incisors in skeletal class II and vice versa in class III [40-42]. Comparing post- to pre-treatment inclination values of lower incisors, Kau et al. reported a significant reduction in skeletal class III, but a significant increase in class I and II [18]. While these findings are in line with our results, changes in the maxillary incisors were not statistically significant [18]. In contrast to our findings, Bhasin et al. described post-treatment a reduction of the pre-treatment proclined incisors in the upper and lower jaw for all skeletal classes, being only significant for skeletal class I in the upper jaw [43].

According to our results, bracket type was negatively correlated with the change in inclination of the lower incisors. Due to the negative correlation, SLB are associated with more proclination than conventional TWIN brackets. Contrary to our findings, other investigations did not find a significant difference in proclination between SLB and conventional brackets during alignment [1, 16, 26, 44] or after treatment [44, 45]. A possible explanation could be, that in our study design the assessment of anterior crowding was not repeated after levelling and alignment, which could have resulted in disregarding some space discrepancies or gaps at T1, affecting the incisors’ inclination. Furthermore, differences in the definition of the second assessment (e.g. insertion before a rectangular arch wire in our study vs. post-treatment [45]) could explain this contradiction.

The remaining variables, which were considered as potential predictors of incisors’ inclination changes, did not reveal statistical significance in any of the three regression equations. Hence, the parameters form of the symphysis (Björk), lip thickness, Angle class, form of malocclusion (overbite), orofacial dysfunction/ habits and headgear do not predict the change in inclination of the incisors during alignment. On the other hand, this observation does not allow the conclusion that these parameters do not influence the incisors’ inclination during alignment. Rather, it can be deduced that these variables are not suitable to significantly contribute to the regression equations. As for the incisors analysed in this study, changes in the mesiodistal angulation of canines and first molars mostly did not differ between Angle class I, II and III [17].

Applying the established regression equations in clinical routine may help the orthodontic practitioner to predict potential (undesired) side effects of levelling and alignment at the front teeth. For example, an excessive proclination of the incisors might increase the risk of gingival recession [4] or result in bite opening with an unfavourable overjet [46]. Considering the pre-treatment values of the individual patient allows for the identification of potentially critical variables in advance, e.g. identifying the risk of increased proclination of the maxillary incisors in case of severe anterior crowding in the upper jaw. Consequently, treatment planning can be modified and individualised to avoid such undesired proclination, e.g. by considering tooth extractions.

### Limitations

Despite the potential benefit of the equations, this study presents some limitations. First, age was not considered in the regression analyses, although the age range of the patients included was broad (9.3–30.0 years) and the time between T0 and T1 was a maximum of two years. Therefore, inclination changes could have been affected by growth. Indeed, age can influence incisors’ inclination [47-49], although variations of the correlation exist among different vertical craniofacial patterns [48, 49], sexes and jaws [49]. Hence, future analyses should consider age as a potential predictor too. Furthermore, due to the retrospective study design clinical data gathered from medical records were collected in the past without the ability to evaluate their reliability and accuracy. Another limitation is the amount of variables regarded as potential predictors. For example, we did not consider the depth of the curve of Spee, the initial lack of space or gaps in the whole jaw and the expansion in the canine, premolar and molar region. Initially, we decided to focus on variables related to the anterior arch due to their close localisation. However, as our findings reveal that more than 50% of the variance in proclination are explained by other, so far unknown factors, future investigations should consider more potential predictors. For example, the (transverse) shape of the arch wire chosen appears to influence incisors’ inclination during alignment [50]. Also, different treatment procedures in cases of tooth agenesis could impact on the post-treatment incisors’ inclination [22]. Despite the above-mentioned limitations, this study contributes to elucidating the understanding of frequently observed changes in the incisors’ inclination during alignment. Compared to previous investigations focusing on few correlation factors [14, 50], this study evaluated the effect of many different potential predictors. Moreover, the sample size recruited in our study was clearly higher than in other analyses [1].

## Conclusions

In conclusion, this study presents three regression equations, predicting the change in incisors’ inclination during levelling and alignment with fixed orthodontic appliances. Among the significant cephalometric and clinical predictors, the amount of anterior crowding and the initial incisors’ inclination of the respective jaw were relevant in all three formulas, showing that more crowding and steeper incisors result in more proclination during alignment. Furthermore, class II elastics predicted only changes in 1-NL, whereas bracket type and skeletal parameters (ANB, individualised ANB, Jarabak ratio) were significant predictors of the lower incisors only.

Applying those equations in treatment planning, an individualised prediction of the expected proclination can be made, allowing for a reduction of undesired inclination changes and associated side effects like gingival recessions. However, further analyses are required to optimise the goodness of fit of the equations.

## Electronic supplementary material

Below is the link to the electronic supplementary material.

Supplementary Material 1

## Data Availability

No datasets were generated or analysed during the current study.

## Abbreviations

SLB: Self-ligating brackets
CI: Confidence interval
