# Supplementary material for: Predictors of changes in incisor inclination during orthodontic levelling and alignment with fixed appliances: a retrospective cross-sectional study
Source: Head Face Med. 2025 May 26;21:41. doi: 10.1186/s13005-025-00519-4 (PMC12105377; doi:10.1186/s13005-025-00519-4)
Supplement: Supplementary file 1 — Supplementary Material 1 [file 13005_2025_519_MOESM1_ESM.docx]

**Supplementary Material “Predictors of changes in incisor inclination during orthodontic levelling and alignment with fixed appliances: a retrospective cross-sectional study”**

**Additional file 1 - table 1:** Descriptive statistics of metric variables. n = number of patients; M= mean; SD= standard deviation; min = minimum; max = maximum, Δ = 1. – 2. lateral cephalogram

| **parameter** | **n** | **M** | **SD** | **min** | **max** |
| --- | --- | --- | --- | --- | --- |
| space discrepancy upper jaw [mm] | 216 | 0.45 | 1.47 | -5.1 | 5.4 |
| space discrepancy lower jaw [mm] | 216 | 0.77 | 1.12 | -2.5 | 5.0 |
| ANB [°] | 216 | 3.42 | 2.21 | -4.6 | 8.7 |
| indiv. ANB [°] | 216 | 3.93 | 1.32 | 0.2 | 6.9 |
| Jarabak ratio [%] | 216 | 66.31 | 4.46 | 56.0 | 81.0 |
| upper lip thickness [mm] | 216 | 12.47 | 2.65 | 6.0 | 21.0 |
| lower lip thickness [mm] | 216 | 14.14 | 2.00 | 9.0 | 21.0 |
| 1. lateral cephalogram (1-1) [°] | 216 | 130.62 | 10.50 | 99.0 | 157.0 |
| 2. lateral cephalogram (1-1) [°] | 216 | 124.19 | 7.85 | 104.0 | 150.0 |
| 1. lateral cephalogram (1-NL) [°] | 216 | 68.85 | 7.51 | 45.0 | 87.0 |
| 2. lateral cephalogram (1-NL) [°] | 216 | 64.88 | 6.21 | 50.0 | 82.0 |
| 1. lateral cephalogram (1-NSL) [°] | 216 | 76.65 | 7.71 | 55.0 | 99.0 |
| 2. lateral cephalogram (1-NSL) [°] | 216 | 72.78 | 6.11 | 54.0 | 88.0 |
| 1. lateral cephalogram (1-ML) [°] | 216 | 85.65 | 6.49 | 70.0 | 103.0 |
| 2. lateral cephalogram (1-ML) [°] | 216 | 83.10 | 6.51 | 63.0 | 100.0 |
| lateral cephalogram $\Delta$ 1-NSL [°] | 216 | 3.87 | 6.32 | -26.0 | 20.0 |
| lateral cephalogram $\Delta$ 1-NL [°] | 216 | 3.98 | 6.39 | -29.0 | 20.0 |
| lateral cephalogram $\Delta$ 1-ML [°] | 216 | 2.56 | 5.25 | -15.0 | 19.0 |
| lateral cephalogram $\Delta$ (1-1) [°] | 216 | 6.43 | 9.30 | -42.0 | 33.0 |

**Additional file 1 - table 2:** Descriptive statistics of clinical categorical variables n= absolute frequency, %= relative frequency

|  |  | **absolute frequency [n]** | **relative frequency [%]** |
| --- | --- | --- | --- |
| **intermaxillary elastics** | no | 191 | 88.4 |
|  | class-II-elastics | 21 | 9.7 |
|  | class-III-elastics | 4 | 1.9 |
|  | overall | 216 | 100.0 |
| **headgear** | no | 181 | 83.8 |
|  | used | 35 | 16.2 |
|  | overall | 216 | 100.0 |
| **swallowing pattern** | somatic | 203 | 94.0 |
|  | visceral | 13 | 6.0 |
|  | overall | 216 | 100.0 |
| **speech disorder** | no | 186 | 86.1 |
|  | yes | 30 | 13.9 |
|  | overall | 216 | 100.0 |
| **mouth- breathing** | no | 212 | 98.1 |
|  | yes | 4 | 1.9 |
|  | overall | 216 | 100.0 |
| **habits** | no | 187 | 86.6 |
|  | yes | 29 | 13.4 |
|  | overall | 216 | 100.0 |

|  |  | **absolute frequency [n]** | **relative frequency [%]** |
| --- | --- | --- | --- |
| **Angle- classification** | I | 79 | 36.6 |
|  | II/1 | 17 | 7.9 |
|  | II/2 | 36 | 16.7 |
|  | II | 65 | 30.1 |
|  | III | 19 | 8.8 |
|  | overall | 216 | 100.0 |
| **form of malocclusion (overbite)** | open bite | 4 | 1.9 |
|  | deep bite | 87 | 40.3 |
|  | regular overbite | 125 | 57.9 |
|  | overall | 216 | 100.0 |
| **Björk`s mandibular structure sign** | clearly anterior (+++) | 3 | 1.4 |
|  | anterior (++) | 10 | 4.6 |
|  | anterior tendency (+) | 84 | 38.9 |
|  | neutral (0) | 79 | 36.6 |
|  | posterior tendency (-) | 32 | 14.8 |
|  | posterior (--) | 7 | 3.2 |
|  | clearly posterior (---) | 1 | 0.5 |
|  | overall | 216 | 100.0 |

**Additional file 1 – table 3:** Descriptive statistics of categorical variables determined with plaster models or lateral cephalograms. n= absolute frequency, %= relative frequency.
